# Bullous Scabies in an Immunocompromised Host

**DOI:** 10.1155/2022/3797745

**Published:** 2022-03-28

**Authors:** James R. Wester, Lesley E Jackson, Kathryn Mokgosi, Tomer Barak, Mahmoud Abu Hazeem

**Affiliations:** ^1^Northwestern University Feinberg School of Medicine, Chicago, IL, USA; ^2^Botswana Global Health Training Program, Beth Israel Deaconess Medical Center, Boston, MA, USA; ^3^Division of Clinical Immunology and Rheumatology, University of Alabama at Birmingham, Birmingham, AL, USA; ^4^Department of Medicine, Scottish Livingstone Hospital, Molepolole, Botswana; ^5^Division of General Internal Medicine, Beth Israel Deaconess Medical Center, Boston, MA, USA

## Abstract

A 40-year-old woman with a history of poorly controlled HIV presented to a district referral hospital in rural Botswana for a generalized skin rash of several months duration. The highly pruritic rash predominantly involved her hands and feet and was associated with bullae that were present for days at a time before rupturing without drainage or discharge. The patient endorsed night sweats, periodic fevers, occasional cough productive of blood-tinged sputum, fatigue, and weight loss. On admission, CD4 count was 46 cells/mm^3^ and viral load was >750000 copies/mL. Pulmonary tuberculosis testing via sputum was negative twice. A blood count demonstrated eosinophilia. Oral acyclovir was started empirically for disseminated herpes virus infection, with topical beclomethasone and intravenous antibiotics for possible superinfected bullous dermatosis. With inadequate response to treatment, a skin biopsy was obtained and microscopic examination demonstrated scabies mites. The absence of skin burrows, the presence of bullae, and working in a low-resource setting without direct access to microscopic examination delayed diagnosis. The patient was initiated on topical permethrin. Oral ivermectin was not available in country and was obtained from overseas shipment, delaying treatment initiation. Drastic improvement was seen after the patient initiated ivermectin. A local nurse in the patient's village visited her community and found multiple individuals with active scabies infection. The patient's discharge was delayed until these community members were treated successfully with topical permethrin. This case describes an atypical presentation of scabies in an under-resourced setting, demonstrating unique diagnostic, therapeutic, and public health challenges.

## 1. Introduction

Crusted scabies, also known as Norwegian scabies, is a parasitic infection of *Sarcoptes scabiei* variety hominis that most commonly occurs in immunocompromised patients [1]. This obligate human parasite invades the patient's epidermis and typically does not travel deeper than the stratum granulosum, which is superficial to the dermis [1]. The female mites create tunnels and lay eggs roughly 15 days after maturation, with larvae then forming within 2-3 days [2]. In classic scabies, approximately 5–15 female mites live on an infected host, whereas in crusted scabies, this number can be as high as hundreds to millions of mites [2].

## 2. Case Presentation

A 40-year-old woman with human immunodeficiency virus (HIV) presented to a district referral hospital in rural Botswana for periodic generalized skin rash of several months duration. The dermatologic flares occurred every few weeks without identifiable triggers, predominantly involving her hands, fingers, and feet bilaterally with involvement of the soles and to a lesser degree her trunk. The exanthem was highly pruritic and associated with skin sloughing and bullae that were present for a few days before their rupture without drainage or discharge. The patient also endorsed night sweats, periodic fevers, cough productive of occasional blood-tinged sputum, fatigue, and weight loss. She visited the local health post multiple times and was prescribed hydrocortisone cream without benefit.

The patient's medical history is notable for HIV for which a highly active antiretroviral therapy regimen of Combivir (CBV) and nevirapine (NVP) was initiated 15 years prior to presentation. The patient stopped taking her HIV medications against medical advice for more than two years. Upon restarting antiretroviral therapy, the patient was transitioned to emtricitabine (FTC), tenofovir (TDF), and dolutegravir (DTG). Approximately 16 months prior to the current presentation, new renal impairment was noted and was thought to be secondary to tenofovir toxicity, so the patient was switched to abacavir (ABC), lamivudine (3 TC), and DTG. However, at presentation, she denied taking any oral medications for several months and for an unspecified amount of time before the rash started. Her CD4 count on admission was 46 cells/mm^3^, and her viral load was >750000 copies/mL. The patient inhaled tobacco regularly and denied alcohol consumption. She occasionally used traditional medicine of unspecified type. She had not been seen in an Infectious Disease Care Clinic (i.e., Botswana's outpatient clinics specifically for people living with HIV) in the past two years.

**Figure 1:**
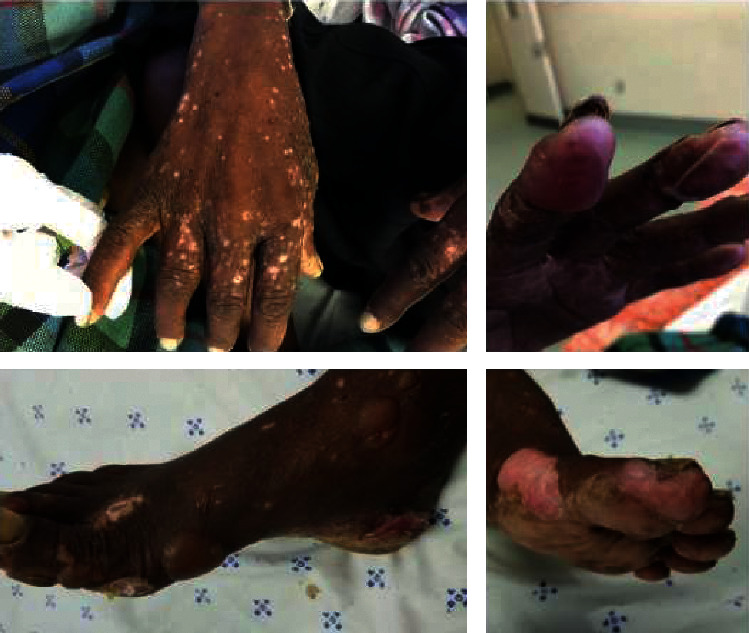
Bilateral distal upper and lower extremities at presentation.

On physical exam, initial vital signs were unremarkable. She was cachectic with signs of malnutrition. Her skin evaluation revealed scaly hyperkeratotic and crusted papules and plaques, as well as tense bullae and erosions affecting the distal upper and lower extremities (Figure 1). The bullae were pruritic and painful and over several days were noted to rupture before being replaced by new bullae. On physical examination, her lungs had fine crepitations in the right lower lung field.

The patient's pulmonary tuberculosis testing at the time of presentation via sputum GeneXpert® MTB-RIF was twice negative. A blood count revealed leukocytosis with worsening eosinophilia over two weeks duration with absolute eosinophil count peaking at 2000 cells/uL, with a reference range of less than 500 cells/uL. A chest X-ray demonstrated a right lower lobe opacity. The patient was treated for community-acquired pneumonia. Additionally, after consulting dermatology, oral acyclovir was started empirically for disseminated herpes virus infection, with topical beclomethasone and intravenous antibiotics for possible superinfected bullous dermatosis (i.e., lichen planus versus bullous pemphigoid versus lichen planus pemphigoid). The patient was also empirically started on trimethoprim/sulfamethoxazole for *Pneumocystis jirovecii* prophylaxis.

With inadequate response to intravenous antibiotics, oral acyclovir, and local steroids, a biopsy/skin scraping with microscopy was obtained during a second dermatology visit. The skin was prepared with mineral oil, and the papule was scraped and examined under a microscope. Examination of the skin scraping revealed scabies mites, thus confirming a diagnosis of crusted scabies in the setting of uncontrolled HIV infection. The absence of the typical appearance of skin burrows, the presence of bullae, and working in low-resource setting without direct access to microscopic examination delayed the diagnosis.

**Figure 2:**
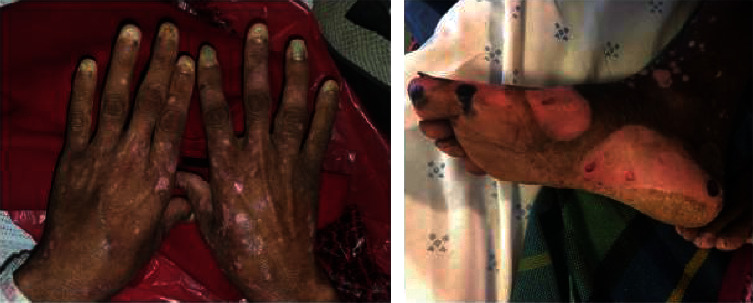
Distal upper and lower extremities after the last dose of ivermectin therapy.

After the diagnosis of crusted scabies was established in our patient, she was initiated on topical permethrin. Oral ivermectin was not readily available in Botswana; once obtained from overseas shipment, it was administered (200 mcg/kg as a single dose on days 1, 2, 8, 9, and 15) with rapid improvement in symptoms and skin appearance (Figure 2). We contacted the local clinic in the patient's village and a local nurse visited the patient's home. Multiple family members were found to have active scabies infection. The patient's discharge was delayed until family members were treated with permethrin.

## 3. Discussion

Though crusted scabies most commonly manifests as hyperkeratotic plaques, diagnostic misclassification is a frequent occurrence due to its diverse range of morphologic presentations [3]. A recent review by Wang et al. demonstrated difficult and often delayed diagnosis of crusted scabies in renal transplant recipients, with frequent misdiagnoses including psoriasis, drug reaction, and allergic and contact dermatitis [4]. Bullous scabies, the rare morphology of scabies identified in this case, has a broad differential including bullous pemphigoid, pemphigus vulgaris, epidermolysis bullosa, bullous impetigo, and contact dermatitis [3, 5, 6]. Additionally, bullous scabies mimics bullous pemphigoid not only from a clinical lens but also histologically as both pathologies have epidermal-dermal separation with dermal eosinophilia [3]. In the presented case, the large number of scabies mites seen microscopically indicated the diagnosis of bullous crusted scabies.

Various mechanisms for the etiology of subepidermal bullae in scabies superinfection have been postulated. Recent literature has suggested that basement membrane zone antigens may be released following mite injury via lytic secretions [7, 8]. These antigens stimulate the production of basement membrane zone antibodies, which attach to the antigen and as a result activate the complement cascade and recruit inflammatory cells [9]. The inflammatory response at the basement membrane leads to the separation at the dermal-epidermal junction resulting in bullae formation.

A common treatment for crusted scabies is topical permethrin, an ovicidal agent which binds to voltage-gated sodium channels in mites and prevents the channels from closing [10]. This continual flow of sodium leads to hyperactivity and thus paralysis and death of the mite [11]. Alternatively, ivermectin is a nonovicidal agent that inhibits ligand-gated chloride channels, stopping nerve conduction and causing paralysis and eventual death of the parasite [10–12]. In the case of classic scabies, treatment with either topical permethrin or topical or oral ivermectin is recommended as a recent Cochrane review failed to detect significant differences in efficacy between these treatments [10]. Guidelines for the treatment of crusted scabies are not uniform internationally. An Australian study published by Currie et al. and the Center for Disease Control and Prevention support the usage of oral ivermectin for a 3–7 dose course in conjunction with topical permethrin [13, 14]. In our particular case, topical permethrin was initially the only available therapy and proved to be an insufficient course of treatment. However, the patient drastically improved after the second dose of ivermectin.

Controlling the spread of transmission presents a public health challenge, particularly in poor socioeconomic conditions and in regions with limited health infrastructure [15, 16]. Past reports have demonstrated the effective control of crusted scabies outbreaks in hospital and community settings utilizing contact tracing, treatment for clinically positive cases, and prophylaxis for direct contacts [17, 18]. Despite diagnostic delay and multiple healthcare workers being exposed to the patient in this case, no healthcare workers developed suspicious or confirmed scabies lesions. Multiple nursing outreach visits to the patient's remote village identified additional cases which were treated with topical permethrin. Prophylactic treatment for direct contacts was not utilized as described in prior studies [17, 18]. In the absence of appropriate public health intervention, our patient likely would have reacquired the infection after returning home. Moreover, more members of the village and surrounding communities would have been at risk of infection. Crusted scabies is a commonly reported phenomena in immunocompromised hosts. However, this case describes an atypical presentation of bullous scabies in an under-resourced setting, demonstrating unique diagnostic, therapeutic, and public health challenges.
